# cGAS–STING signalling in cancer: striking a balance with chromosomal instability

**DOI:** 10.1042/BST20220838

**Published:** 2023-03-06

**Authors:** Bruno Beernaert, Eileen E. Parkes

**Affiliations:** Department of Oncology, University of Oxford, Old Road Campus Research Building, Oxford OX3 7DQ, U.K.

**Keywords:** cGAS-STING, chromosomal instability, DNA synthesis and repair, immunosurveillance, innate immunity

## Abstract

Chromosomal instability (CIN) is a hallmark of cancer that drives tumour evolution. It is now recognised that CIN in cancer leads to the constitutive production of misplaced DNA in the form of micronuclei and chromatin bridges. These structures are detected by the nucleic acid sensor cGAS, leading to the production of the second messenger 2′3′-cGAMP and activation of the critical hub of innate immune signalling STING. Activation of this immune pathway should instigate the influx and activation of immune cells, resulting in the eradication of cancer cells. That this does not universally occur in the context of CIN remains an unanswered paradox in cancer. Instead, CIN-high cancers are notably adept at immune evasion and are highly metastatic with typically poor outcomes. In this review, we discuss the diverse facets of the cGAS–STING signalling pathway, including emerging roles in homeostatic processes and their intersection with genome stability regulation, its role as a driver of chronic pro-tumour inflammation, and crosstalk with the tumour microenvironment, which may collectively underlie its apparent maintenance in cancers. A better understanding of the mechanisms whereby this immune surveillance pathway is commandeered by chromosomally unstable cancers is critical to the identification of new vulnerabilities for therapeutic exploitation.

## Introduction: CIN as a driver of immunostimulatory genomic DNA

**Figure 1.**
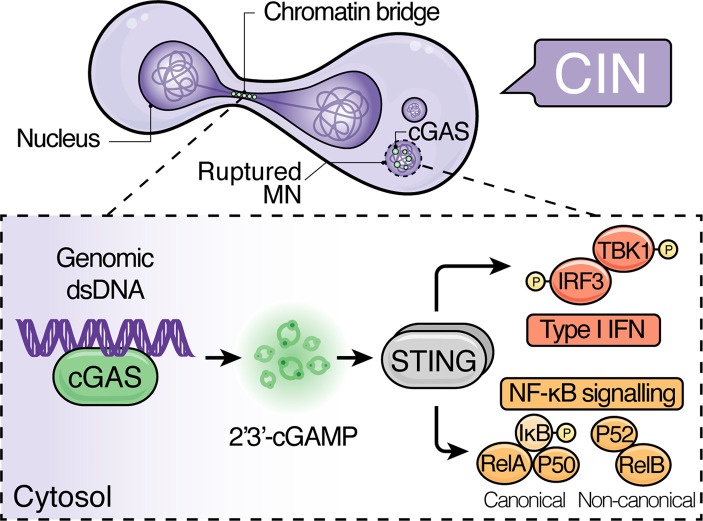
CIN as a driver of cGAS–STING signalling. Chromosome segregation errors in chromosomally unstable cells can lead to the generation of immunostimulatory genomic dsDNA in the form of micronuclear DNA that becomes exposed to the cytosol upon MN rupture or stretched chromatin in chromatin bridges. Production of 2′3′-cGAMP by cGAS activates STING, which, in turn, activates TBK1. TBK1 phosphorylates the transcription factor IRF3, driving the expression of several type I IFNs. Besides TBK1/IRF3/IFN signalling, STING also instigates NF-κB signalling programs. Canonical NF-κB signalling is triggered through the phosphorylation and suppression of NF-κB inhibitor, alpha (IκBα), releasing the RelA/p50 complex into the nucleus. STING also elicits non-canonical NF-κB signalling through the p100/RelB complex through an as yet poorly understood mechanism. CIN, chromosomal instability; dsDNA, double-stranded DNA; IFN, interferon; MN, micronucleus.

Chromosomal instability, characterised by persistent errors in chromosome segregation, is a hallmark of cancer that drives tumour evolution [1]. Chromosome segregation errors frequently lead to the formation of structures known as micronuclei (MN) — which arise from whole chromosomes or fragments that lag behind during anaphase — and chromatin bridges [2]. Upon mitotic exit, mis-segregated chromosomes recruit their own nuclear envelope, which is often structurally unstable, leading to MN envelope collapse and exposure of micronuclear contents to the cytosol [3] (Figure 1).

The cyclic GMP-AMP synthase (cGAS) is the principal sensor of pathogen-derived cytosolic double-stranded DNA (dsDNA) of infected host cells in mammals [4–6]. However, owing to its mechanism of activation, which relies on the detection of ‘out-of-context’ cytosolic dsDNA rather than pathogen-specific DNA sequences and features [7], misplaced ‘self-DNA’ arising from ruptured MN [8–10] and chromatin bridges [11] is also recognised by cGAS, linking CIN to innate immunity [8–10, 12, 13]. Binding of cGAS to dsDNA activates its catalytic activity and triggers the production of the cyclic dinucleotide 2′3′-cGAMP, which activates the immune signalling hub, stimulator of interferon genes (STING) [4–6]. Upon activation, STING relocalises from the endoplasmic reticulum to the perinuclear Golgi apparatus, where it forms an oligomeric platform on which TANK-binding kinase 1 (TBK1) phosphorylates the transcription factor interferon regulatory factor 3 (IRF3), prompting its nuclear entry and the expression of genes encoding type I interferons (type I IFNs) [14]. STING relocation also activates the canonical and non-canonical nuclear factor kappa-light-chain-enhancer of activated B cells (NF-κB) pathways [15], which drive the expression of pro-inflammatory genes encoding a swathe of pleiotropic cytokines and chemokines [16].

Importantly, whilst STING-driven IRF3- and NF-κB-dependent signalling cascades have overlapping functions with regards to mounting cell-intrinsic innate immune responses, their differential regulation downstream of STING, as well as the chronicity and intensity of signalling are major determinants of cGAS–STING pathway output as either anti- or pro-tumorigenic [17]. While it is evident that stimulation, particularly in the acute setting, of STING in antigen-presenting cells is tumour suppressive [18–20], the role of cGAS–STING signalling as a result of constitutive CIN-driven stimulation in the tumour environment is not as clearly defined. Indeed, recent evidence instead favours a role for cGAS–STING in the progression of chromosomally unstable cancers [21].

## cGAS and STING are maintained across tumours

The near-ubiquitous presence of varying degrees of CIN among cancers [1] suggests that tumour cells experience selective pressures that should compel them to overcome cGAS–STING activation and immune surveillance. Indeed, that cGAS–STING activity can represent a barrier to tumorigenesis has been demonstrated experimentally *in vivo*, including in murine models of colitis-associated colon cancer [22–24], and inferred from observations that cGAS and STING are the subject of loss-of-function mutations and/or epigenetic silencing in a variety of cancers [25], including subsets of glioma [26], colorectal [27], melanoma [28, 29], gastric [30], lung [31] and ovarian [32] cancers. Nevertheless, mutations are rare — with a mutational frequency of just 0.6% and 0.5% for cGAS and STING, respectively, among tumours of The Cancer Genome Atlas (TCGA) PanCancer database [1] — and the extent of epigenetic silencing demonstrates a large variation in cGAS and STING promoter methylation in tumour samples compared with corresponding normal tissues. Indeed, in multiple tumour types, including upper gastrointestinal, colorectal, bladder and thyroid cancers, average cGAS–STING promoter methylation is decreased, with a concomitant increase in expression, in tumour over normal tissue [25, 33], suggesting that direct silencing of cGAS and STING is not a pervasive mechanism for immune evasion in cancers.

To the contrary, emerging evidence from multiple tumour settings, including carcinogen-induced skin cancer [34], Lewis lung carcinoma [35], chromosomally unstable triple-negative breast cancer (TNBC) [36] and metastatic lung and breast cancer models [13, 37] has now begun to reveal a role for cGAS–STING activity as a driver of tumour progression.

## Cell-intrinsic functions of cGAS–STING in CIN: genome stability regulation

**Figure 2.**
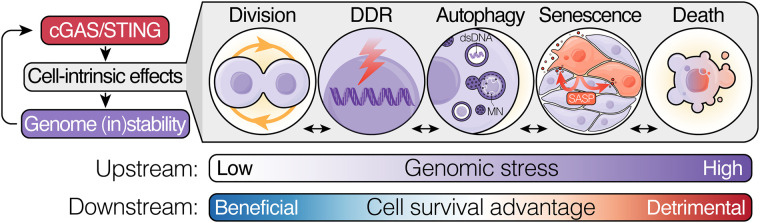
Cell-intrinsic functions of cGAS and STING in genome stability regulation. cGAS and STING (both inter- and independently) exert influence over several signalling programs for the regulation of genome stability and cell survival outcomes downstream of CIN. Regulatory functions of cGAS–STING can be beneficial to cell survival and chromosomal stability in the context of lower levels of genomic stress, supporting orderly cell division, DNA repair responses and the autophagic clearance of cytosolic DNA. However, as genotoxic stress increases, cGAS–STING activity skews increasingly towards genome-destabilising, as well as anti-proliferative and pro-apoptotic signalling programs, such as DDR inhibition, senescence and autophagic and mitotic death, pushing cells past the tolerable CIN threshold and restricting the propagation of cells with excessively unstable genomes. DDR, DNA damage response; dsDNA, double-stranded DNA; MN, micronucleus; SASP, senescence-associated secretory phenotype.

The cGAS–STING pathway has mainly been examined through the lens of its tumour cell-extrinsic function: namely, the activation of cell-mediated immunity through type I IFN signalling [20]. However, emerging evidence suggest that there are myriad cellular processes beyond cytosolic immunity in which cGAS and STING are active participants, which may explain why pathway activity is maintained in some tumour contexts. Moreover, the convergence of multiple of these processes, including DNA damage responses, cell cycle control, senescence, autophagy and cell death, on genome stability regulation underscores the relevance of cell-intrinsic cGAS and STING functions to CIN tumours in particular (Figure 2).

### DNA damage responses

#### Immune-dependent

The role of cGAS–STING-driven immune surveillance as a cell-extrinsic genome surveillance mechanism downstream of DNA damage-induced CIN has been well-established [8, 9, 12, 38]. However, components of the cGAS–STING pathway have been implicated in more direct modes of genome surveillance in DNA damage contexts.

For example, several studies have shown that the expression of a subset of interferon-stimulated genes (ISGs), known as the IFN-related DNA damage resistance signature (IRDS), which is associated with resistance to DNA-damaging chemo- and radiotherapies [39–41] is sustained by cGAS–STING-driven IFN activation following prolonged DNA damage [42–44]. Although the full extent of mechanisms underlying the protective nature of certain chronic IFN responses remains unknown, two studies linked persistent STING activity with the IFN-driven expression of several *PARP*-family genes, including *PARP9* and *PARP12,* posited to promote tumour cell resistance to genotoxic stresses [43, 45] by enhancing DNA damage repair responses [46].

Whilst this supports that subsets of cGAS–STING-driven ISGs can exert genome-stabilising effects that mediate resistance to some genotoxic stresses, genome de-stabilising functions have also been reported. One such instance involves the ubiquitin-like modifier ISG15, an important downstream target of STING-driven IFN [47]. Up-regulation of ISG15 is reported to accelerate DNA replication fork progression by promoting the fork restart activity of the DNA helicase RECQ1 after fork stalling, resulting in extensive DNA damage and chromosomal aberrations, sensitising cancer cells to chemotherapeutic treatments [48]. However, a number of genome-stabilising functions have also been ascribed to ISG15 [49–52], in keeping with its described function as a transcriptional target of p53 [53], exemplifying the broader paradox of innate immune responses in the modulation of the DNA damage response (DDR) [54].

The role of innate immune signalling in DDR regulation extends beyond STING-driven IFN responses, with several reports of canonical and non-canonical NF-κB signalling pathway involvement in double-strand break (DSB) repair [55–59]. However, notwithstanding the well-established role of NF-κB pathways as downstream effector programs of the cGAS–STING axis [15], a direct participation of cGAS–STING was not investigated in these reports.

Despite the differential impact of cGAS–STING-dependent responses on genome stability, these studies collectively demonstrate the centrality of cGAS–STING signalling, not only as a consequence of CIN, but as an active regulator of DNA damage responses.

#### Immune-independent

Multiple emerging mechanisms of DDR modulation by cGAS–STING pathway components have also been described to occur in a manner that is immune signalling or interferon independent. Intriguingly, cGAMP-induced activation of the DDR has recently been described in cells from invertebrate species that lack IRF3 and type I interferons, suggesting that involvement of the cGAS–STING axis in DDR signalling predates the evolution of the type I interferon system in vertebrates [60].

One such mechanism, reported by Banerjee et al. [60], involves the cGAS/STING/TBK1 axis, but not its downstream canonical IFN signalling pathway, in mediating DDR signalling following treatment with genotoxic agents [60]. Mechanistically, cGAS–STING-activated TBK1 was shown to promote the autophosphorylation of the DDR kinase ATM, resulting in the engagement of the CHK2–p53–p21 transduction pathway and the consequent enforcement of a G_1_ cell cycle arrest.

Other immune-independent functions for cGAS–STING in DDR regulation have been ascribed to non-canonical functions of cGAS that pertain to its capacity to enter the nucleus and associate with chromatin [61]. For instance, two independent studies recently found that nuclear cGAS can act as a suppressor of homologous recombination (HR)-mediated DSB repair, albeit through distinct proposed mechanisms [62, 63]. In the model proposed by Liu et al. [62], cGAS is recruited to DSBs, where it interacts with PARP1 via poly(ADP-ribose) moieties, impeding the formation of the PARP1-Timeless complex, required for efficient HR [62]. Jiang et al. [63], on the other hand, proposed a more indirect mode of HR regulation, whereby cGAS oligomerisation compacts the dsDNA template into a higher-order state that is less accessible to RAD51-mediated strand invasion [63]. Although distinct in mechanistic terms, functionally both studies showed that cGAS exacerbates DNA damage and accelerates CIN, promoting tumorigenesis [62] or precipitating cell death in cells exposed to acute genomic stress [63].

Conversely, two recent studies ascribed genome-stabilising effects to nuclear cGAS with respect to its ability to mediate responses to DNA damage. In the first of these, recruitment of cGAS to replication forks was shown to decelerate fork progression to safeguard genome stability. More specifically, loss of cGAS was shown to result in excessive fork speed and premature fork restart after fork stalling, eliciting the activation of the DDR sensor ATR and sensitising cells to DNA-damaging treatments [64]. In a second report, cGAS was observed to occupy deprotected telomeres during mitosis, where it was proposed to repress the DNA damage repair signalling activity that gives rise to CIN-initiating chromosome end-to-end fusions [65].

Of note, the above-described functions of nuclear cGAS were shown to be independent of STING when investigated [63–65], suggesting that cGAS and STING integrate distinct functions for the regulation of DNA damage responses and genome stability. Indeed, STING itself has also been proposed to act in a genome stabilising-manner, independently of cGAS. For example, a study in breast cancer cells identified that part of the intracellular STING pool resides at the inner nuclear membrane, acting as a positive regulator of the DDR and shielding against excessive genomic instability separately from both cGAS and downstream interferon signalling [66].

Altogether, these studies further add to the notion that the increasingly diverse spread in reports surrounding the localisations of cGAS and STING and their proposed inter- and independent functions reflect an array of roles in various homeostatic processes that intersect with cell-intrinsic genome stability regulation.

### Cell division & senescence

This notion is further bolstered by recent studies implicating cGAS and/or STING and their downstream transcriptional programs in cell division regulation [64, 67, 68]. In one such report, STING was found to drive the p53- and NF-κB-dependent accumulation of the cyclin-dependent kinase inhibitor p21, ensuring timely entry to S-phase and mitosis [67]. Loss of STING, but not cGAS, promoted precocious cell division, resulting in the acquisition of chromosomal aberrations and CIN, which was further enhanced by ionising radiation. A similar requirement for STING, as well as cGAS, in regulating p21 levels, was recently also found to impede premature mitotic entry and resultant chromosome segregation errors through the IRF3-driven transcriptional up-regulation of p53 [68], suggesting that multiple components downstream of STING, including the IRF3 and NF-κB signalling arms, converge on the regulation of cell division to maintain genome stability.

However, control of cGAS–STING over the cell cycle extends beyond the maintenance of chromosomal homeostasis during routine cell division and also plays a key role in establishing a state of permanent cell-cycle arrest, known as cellular senescence, in response to persistent sub-lethal genomic stress [69]. The cytosolic DNA species that are generated in such stress contexts are recognised by cGAS, instigating the STING-mediated production of multiple cytokines and chemokines [70–72], collectively referred to as the senescence-associated secretory phenotype (SASP), which reinforces and spreads the senescent growth arrest phenotype in autocrine and paracrine manners, respectively [73–76]. As such, cGAS and STING can co-operate to ensure segregation error-free cell division at steady state and prevent the expansion of cells with excessively unstable genomes.

### Autophagy

The macroautophagic pathway (autophagy) is a strictly regulated pathway that oversees the degradation of excess or dysfunctional cytosolic components, and is therefore tightly interrelated with genome integrity [77]. Indeed, CIN and aneuploidy — a phenotypic outcome of CIN [78] — are associated with an increase in autophagic activity [79–81] and endow cells with a selective vulnerability to the suppression of autophagy [82, 83], suggesting that autophagic activity attenuates the deleterious effects of CIN-associated stresses.

A similarly mutual relationship has emerged between autophagy and the cGAS–STING pathway, with multiple reports highlighting the role of autophagy in tuning STING responses through the degradation of multiple components of the cGAS–STING axis, including cGAS and STING themselves, as well as its cytosolic dsDNA substrates [84–91]. Crucially, cGAS–STING was recently shown to play an evolutionarily conserved role in autophagy activation, independently of downstream interferon induction, suggesting that it constitutes a primordial effector function of the STING pathway [92].

Moreover, cGAS–STING-driven autophagy has been shown to mediate the clearance of free endogenous cytosolic dsDNA upon both chromosome gains [93] and DNA damage [92], analogous to its initially reported role in the clearance of pathogenic cytosolic DNA to prevent persistent immune signalling [87]. Together with other observations of autophagic clearance of cytosolic genomic dsDNA in genomic stress contexts [94–96], this suggests that cGAS–STING-driven clearance of cytosolic genomic DNA may represent a tolerogenic mechanism for genomic instability. In line with this, cGAS-dependent autophagy activation was recently reported to promote the growth and survival of highly chromosomally unstable BT-549 TNBC cells, but not of breast cancer lines with lower cytosolic genomic DNA burdens, by enabling the clearance of DNA from their cytosol [97].

Autophagic degradation has also been observed to mediate the removal of micronuclei [10, 98]. Since MN not only constitute a passive marker of CIN, but can actively fuel it through their reincorporation into primary nuclei [99, 100], their autophagic clearance may thus have a directly genome-stabilising effect, echoing early reports highlighting the genome-stabilising nature of autophagy [101, 102]. Intriguingly, a direct role between autophagic clearance of MN and cGAS was recently reported by Zhao et al. [103], who showed that micronuclear cGAS interacts with the autophagy mediator LC3B to help target MN to lysosomes for degradation [103]. Strikingly, the capacity of cGAS to act as a ‘MN-phagy’ receptor was found to be independent of both its cGAMP enzymatic activity and DNA-binding capacity, suggesting that it represents a function that is distinct from its role as a driver of STING-dependent autophagy upon cytosolic DNA sensing [87, 92, 104].

### Cell death

Despite the cytoprotective role of autophagy in certain stress contexts, its hyperactivation can also lead to cell death [105]. Indeed, a recent study showed that in cells that bypass senescence (in the absence of p53 and Rb) and reach replicative crisis, extensive telomeric DNA damage leads to the generation of cytosolic DNA species that drive autophagic cell death in a cGAS–STING-dependent manner [106]. Interestingly, attenuation of autophagy or the cGAS–STING pathway enabled cells to bypass crisis despite the accumulation of gross karyotypic aberrations, suggesting that cGAS–STING-driven autophagic death is an essential safeguard against genomic instability driven by telomere crisis. The observation that cGAS–STING-driven autophagic death also occurs outside of telomere crisis in response to cell–cell fusion — a catastrophic event that precipitates aberrant mitosis and CIN — suggests it may constitute a conserved tumour-suppressive mechanism for the elimination of cells experiencing catastrophic levels of CIN [107], despite reports of a protective role for autophagy in less severe CIN contexts.

As such, cGAS–STING-driven autophagy may, to an extent, act on a cell-intrinsic level to ensure unstable genome homeostasis by enabling cell survival at low-level CIN and driving the elimination of tumour cells in circumstances of high or acutely-driven CIN. Since autophagy is also critical for mediating cell-extrinsic homeostatic effects through its involvement in multiple contrasting facets of immunity, including innate immune signalling and danger signal emission (i.e. adjuvanticity), as well as tumour cell antigenicity [108, 109], it is possible that its control through cGAS–STING represents another avenue through which tumours may interface with the local microenvironment (discussed below). However, given the contradictory nature of autophagy in immunity, it remains unclear how such tumour cell-extrinsic effects of STING-driven autophagy may influence tumour cell survival. Nonetheless, it is tempting to speculate that they may act in a manner akin to its cell-intrinsic effects: enabling survival and maintaining tumour growth by suppressing immune recognition at lower-level CIN and stimulating immune responses through the release of danger signals and antigens from dead and dying cells in high-CIN settings.

cGAS–STING activity appears to drive similarly paradoxical outcomes with respect to its function in cell division. For instance, while cGAS–STING pathway activity slows mitosis to avoid error-prone premature cell division under baseline conditions [67, 68], it can also instigate mitotic death in an IRF3 activity-dependent, yet transcription-independent, manner in cells undergoing a prolonged arrest in mitosis [110]. Thus, the strength and duration of activating signals — emerging determinants of how cGAS–STING-signalling outcomes are shaped in different immune cell contexts [17, 111, 112] — also appear to dictate signalling outcomes in CIN contexts.

## cGAS–STING as a driver of chronic pro-tumour inflammation in CIN tumours

**Figure 3.**
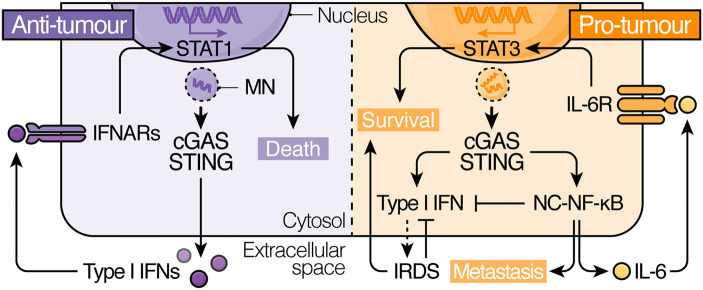
The dichotomous role of CIN-driven cell-intrinsic cGAS–STING inflammatory signalling in cancer. Activation of cGAS–STING-dependent inflammatory signalling downstream of CIN can exert both tumour-suppressive, as well as tumour-promoting effects in a cell-intrinsic manner. Expression of type I IFNs and downstream engagement of IFNAR/STAT1 induces the expression of multiple effectors, including pro-apoptotic and anti-proliferative genes, that are detrimental to tumour cell survival. Conversely, cGAS–STING-dependent activation of NC-NF-κB signalling can drive IL6/STAT3 signalling and EMT programs, which promote tumour growth and metastasis, respectively. Induction of protective pathways associated with chronic cGAS–STING activity, such as the IRDS and NC-NF-κB signalling can also directly antagonise type I IFN activation, dampening the overtly tumour-suppressive effects of cGAS–STING. IFN, interferon; IFNAR, IFN receptor; IL-6R, IL-6 receptor; IRDS, IFN-related DNA damage resistance signature; MN, micronucleus; NC-NF-κB, non-canonical NF-κB.

Although the emerging link between genome stability regulation and the non-canonical functions of cGAS–STING may explain how some chromosomally unstable tumours may derive benefits from STING pathway maintenance, it does not explain how it enables tumours to overcome the high cost of immune surveillance that is described to oftentimes ensue CIN-driven inflammatory signalling [8, 9, 12, 38]. Strikingly, mounting evidence suggests that continuous activation of the cGAS–STING pathway as a result of CIN can fuel chronic inflammation which favours rather than limits the formation, progression and spread of tumours [113, 114], suggesting that tumours harbouring unstable genomes are able to de-emphasise overtly anti-tumour signalling outcomes downstream of STING in favour of more pro-tumour ones (Figure 3).

Using transplantable metastatic tumour models of TNBC, Bakhoum, Ngo et al. [13] recently demonstrated that CIN enriches tumour cells for epithelial–mesenchymal transition (EMT)-related gene expression, supporting metastatic dissemination, in a cGAS–STING-dependent manner [13]. Strikingly, rather than activating canonical NF-κB or type I IFN signalling, cGAS–STING activity in CIN-high TNBC cells was shown to preferentially activate the metastasis-promoting non-canonical NF-κB (NC-NF-κB) pathway [115, 116], suggesting that tumour cells can rewire their cGAS–STING signalling circuitry to co-opt its pro-tumour signalling arms. Although it is unclear how universal a metastatic escape route this represents across different tumour types, CIN has also recently been reported to fuel cGAS–STING-dependent pro-metastatic inflammation in subsets of uveal melanoma (UM) [117] and pancreatic ductal adenocarcinoma (PDAC) [118], besides aggressive breast cancer [13].

Collectively, these studies suggest that CIN-driven activation of cGAS–STING is an important driver of tumour cell invasiveness and metastasis. However, invasiveness is not an obligate outcome of CIN. For instance, Vasudevan et al. [119] recently showed that acute induction of CIN suppresses rather than promotes invasive behaviour across several cancer and non-cancer cell lines, despite inducing extensive micronucleation and NC-NF-κB and inflammatory signal activation [119]. How these discrepancies can be reconciled is unclear, but understanding in what contexts cGAS–STING produces pro-metastatic versus anti-tumour outcomes will be crucial to the appropriate administration of therapies that converge on STING pathway activation.

The tumour cell-intrinsic benefits of CIN-driven inflammation extend beyond its enhancement of metastatic behaviour in advanced stage tumours and have recently also been implicated in early tumour development. For instance, Hong et al. [36] recently showed that some CIN-high primary tumours rely on a pro-survival cGAS–STING-dependent inflammatory response to surmount CIN-imposed stresses [36]. *In vitro* work showed that loss of cGAS and STING sensitised TNBC cells to both acute (chemical-induced) and chronic (genetic) induction of chromosome mis-segregation and was rescued by the additional abrogation of STAT1, indicating a largely STAT1-driven sensitivity to CIN in their model system. As CIN induction was shown to trigger IL-6 expression and the engagement of the pro-survival IL-6/JAK/STAT3 signalling axis [120, 121] in a cGAS–STING-dependent manner, it was posited that its activation may constitute a requirement to overcome the deleterious effects of CIN-induced STAT1. Moreover, loss of cGAS and STING, as well as inhibition of the IL-6 receptor (IL-6R) were shown to specifically impair the outgrowth of chromosomally unstable TNBC tumours *in vivo*, suggesting that CIN imparts a tumour cell-intrinsic — if not an additional cell-extrinsic — dependency on cGAS–STING-driven IL-6/STAT3 signalling in primary TNBC cancers.

A similar reliance on cell-intrinsic cGAS–STING-driven inflammation for *in vivo* tumour growth was also recently reported for human and murine breast tumour cells exhibiting genomic instability due to the overexpression of MYO10, an unconventional myosin that is up-regulated in several aggressive cancers [122]. Interestingly, in this report, STING depletion was shown to suppress the acceleration in tumour growth seen to occur upon CIN induction to the growth rate observed in ‘genomically stable’ tumours, rather than curbing growth altogether, as reported above [36]. This implies that, whereas in some CIN contexts, cGAS–STING-driven inflammation may help cancer cells survive by counterbalancing the costs of genome instability, in other settings, it explicitly lets them thrive.

Altogether, these studies suggest that CIN-driven cGAS–STING inflammatory signalling may be tolerated and even co-opted by tumours that are able to shunt pathway activity towards pro-tumour signalling programs, such as the NC-NF-κB and STAT3 arms of the cGAS–STING pathway, rather than more outrightly anti-tumour arms, such as type I IFN receptor (IFNAR)-driven STAT1 signalling. Moreover, emerging evidence suggests that activation of pro-survival immune signalling arms downstream of cGAS–STING, such as NC-NF-κB signalling and the IRDS, may even directly antagonise the induction of its anti-tumour arms [44, 123, 124], implying that some tumour cells may become addicted to the protective effects of cGAS–STING responses in the face of persistent CIN.

**Figure 4.**
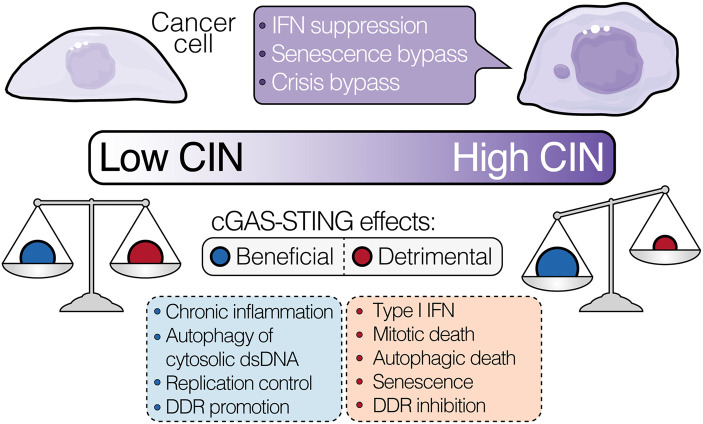
Genetic context and degree of CIN dictate cGAS–STING signalling outcomes. The cGAS–STING pathway triggers multiple signalling programs in a cell-autonomous manner, which can produce opposing effects on tumour cell fitness. Whether engagement of the cGAS–STING axis results in an outcome that is beneficial or detrimental to tumour cell survival depends on the cumulative status of its downstream pathway components. Highly chromosomally unstable tumours likely acquire adaptations that enable them to avoid activation of overtly anti-proliferative and/or pro-apoptotic signalling programs, such as the senescence program, telomere crisis and type I IFN signalling, and further enhance the beneficial effects of pro-tumour programs. In addition, the fitness costs imparted by CIN-associated stresses may increase the reliance of high CIN tumours on the genome-stabilising effects and pro-survival programs induced by cGAS–STING. As such, chromosomally unstable cancers may tip the balance between beneficial and detrimental cGAS–STING signalling outcomes downstream of CIN in their favour. CIN, chromosomal instability; DDR, DNA damage response; dsDNA, double-stranded DNA; IFN, interferon.

The past few years have seen a shift in our understanding of cGAS–STING signalling in cancer. It is becoming increasingly clear that cGAS–STING pathway functions in tumours extend beyond the induction of tumour-suppressive inflammation. Indeed, multiple studies now point to cGAS–STING pathway involvement in multiple cell-intrinsic signalling programs (as outlined above) that can exert opposing outcomes on tumour cell fitness. Hence, whether cGAS–STING engagement results in an outcome that is beneficial or detrimental to tumour cell survival depends on the status of each of its downstream pathway components (Figure 4). As chromosomally unstable cancers are under especially high selective pressure to acquire adaptations that help avoid tumour-suppressive cGAS–STING-driven programs (e.g. senescence, type I IFN signalling and autophagic death upon crisis) [1, 125], they may disproportionately skew pathway activity towards pro-survival outcomes that further tumour progression.

## cGAS–STING as an orchestrator of the pro-tumour microenvironment

**Figure 5.**
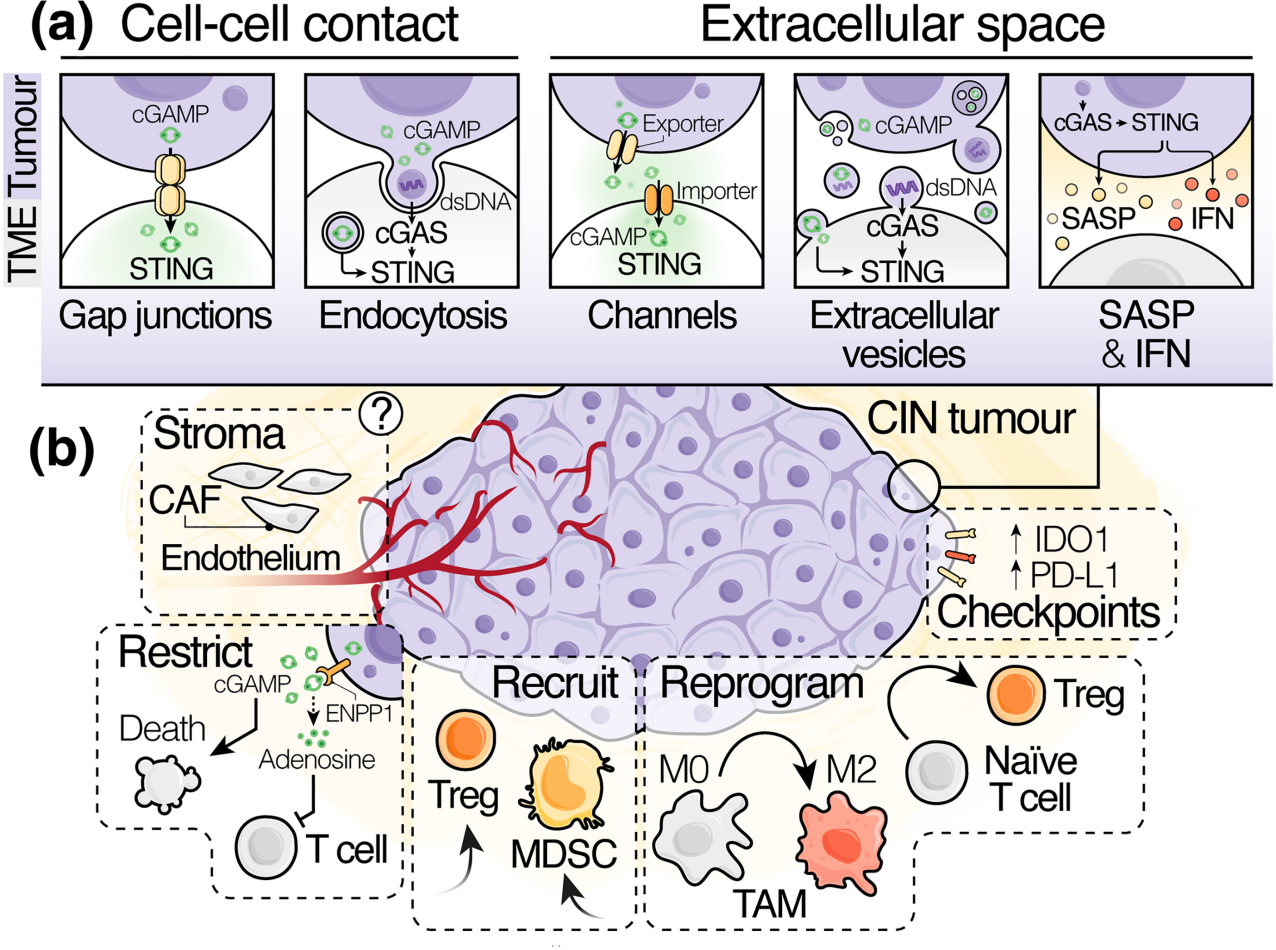
The pro-tumour functions of cGAS–STING signal transmission in the TME. (**a**) Intercellular routes through which mediators of cGAS–STING signalling, predominantly in the form of cGAMP or dsDNA, can be propagated into neighbouring (via cell–cell contacts) or more distant cells (via the extracellular space) of the TME to shape the tumour landscape. (**b**) Reported functions of cGAS–STING signalling in the establishment of an immune-suppressive pro-tumour TME which may be at play in CIN tumour settings. CAF, cancer-associated fibroblast; CIN, chromosomal instability; dsDNA, double-stranded DNA; IFN, interferon; MDSC, myeloid-derived suppressor cell; SASP, senescence-associated secretory phenotype; TAM, tumour-associated macrophage; TME, tumour microenvironment; Treg, regulatory T cell.

The full scope of pro-tumorigenic cGAS–STING functions likely involves the concerted action of multiple components of the tumour microenvironment (TME), beyond cancer cells. Indeed, many studies have ascribed the influence of cGAS–STING on tumour development and metastasis to the activation of STING signalling in multiple cellular components of the TME other than tumour cells, including astrocytes [37], mesenchymal stromal cells [126] and phagocytes [34]. This propagation of STING signalling into the TME is enabled by a complex network of intercellular transport routes that comprises several cell type-specific channels [127–129], transporters [130–133], gap junctions [37, 134–137] and extracellular vesicles (EVs) [138], as well as endocytic routes, such as phagocytosis [139–141] and transcytosis [142] (Figure 5a).

Collectively, these routes help transmit cGAS–STING signal mediators, such as cGAMP and dsDNA, from chromosomally unstable tumour cells to neighbouring bystander cells via direct cell–cell contacts or across the extracellular space to more distant cells [143]. As such, cumulative STING activity within tumours is not just determined by the intrinsic cytosolic DNA burden and the expression of cGAS–STING components within cancer cells, but also by the composition of the TME and the capacity of its distinct cellular components to import and respond to cGAS–STING ligands (Figure 5b). This not only permits cGAS–STING activation within the TME of CIN-high tumours with defective DNA sensing pathways, but also offers ample opportunity for cancer cells to intercept or fine-tune cGAS–STING signal transmissions to their benefit. A notable example of this involves the breakdown of extracellular cGAMP by the cGAMP hydrolase ENPP1 [144, 145], which can drive the accumulation of the immune-suppressive metabolite adenosine [146], resulting in decreased immune infiltration and increased metastasis in CIN tumours [147].

Whilst additional mechanisms, such as the digestion of chromatin by the secreted deoxyribonuclease (DNase) I-family nucleases DNase I and DNase I-like 3 (DNase IL3), have been proposed to limit extracellular cGAS substrate availability [148, 149], it is unclear whether and to what extent they restrain cGAS–STING signal transmission to the TME by CIN tumours.

Whether chronic STING activation in tumours necessarily fosters a pro-tumorigenic TME remains unclear. A pan-cancer correlative analysis of TCGA datasets looking at the association between STING mRNA expression and immune cell infiltration revealed that STING expression correlates positively with infiltration of most immune cell types across cancers, rather than any one subtype [150]. This reflects that rather than recruiting specific subsets of cytotoxic or immunosuppressive immune cells into the tumour, STING exerts a broadly chemo-attractive effect, which may underlie its paradoxical role in the shaping of tumour landscapes. Indeed, aside from its well-established involvement in promoting anti-tumour immune responses [20], increasing evidence indicates that STING activity also plays a role in establishing a tolerogenic tumour microenvironment.

STING activity has been reported to promote the recruitment and/or induction of immune-suppressive myeloid-derived suppressor cell (MDSC) [35, 151–153] and regulatory T cell (Treg) [33, 154] populations, as well as the polarisation of monocytes to immune-suppressive ‘M2-like’ tumour-associated macrophages [155, 156]. In addition, chronic or over-stimulation of STING has been shown to selectively initiate anti-proliferative and pro-apoptotic programs in T cells [111, 129, 157–161], which, combined with the STING/IFN-dependent up-regulation of immune checkpoints, such as IDO1 and PD-L1, in the TME [12, 35, 162–167] may limit the overall anti-tumour potency of the T cell compartment.

As such, whilst transient STING induction appears favourable to anti-tumour immunity, prolonged activation can elicit immunoregulatory mechanisms that help tumours avoid immune-mediated destruction. Indeed, several correlative pan-cancer studies have related the degree of aneuploidy and CIN to increased markers of immune evasion and a decreased cytotoxic immune infiltrate [168–170], suggesting a role for CIN, albeit not necessarily CIN-driven cGAS–STING, in driving immunosuppression. However, the functional interaction between chronic cGAS–STING activation as a consequence of CIN and the tumour landscape has not been closely examined.

Crucially, aside from immune cells, cells of the non-immune stromal compartment, such as cancer-associated fibroblasts (CAFs) and endothelial cells, also act as key mediators and effectors of cGAS–STING signalling in the TME [142, 171–174]. However, despite evidence that higher tumour aneuploidy correlates with increased non-immune stromal fractions across several solid tumours [169], potential interactions between CIN-driven STING signalling and such stromal components remain unexplored. Further investigation of this and other aspects of cGAS–STING signalling in distinct compartments of the tumour microenvironment is likely to provide valuable insight enabling tailored targeting of cGAS–STING-driven signalling.

## Conclusion

It is becoming increasingly clear that the outcomes of cell-intrinsic cGAS–STING-driven programs in CIN contexts are paradoxical in nature. Whereas in homeostatic and low genomic stress conditions they appear to promote survival mechanisms and genome stability, in excessively high CIN settings they can amplify CIN to catastrophic levels and bring about cell death (Figure 2). As such, cGAS–STING-driven genome-stabilising functions (e.g. DDR promotion, cell division control, autophagy) and anti-proliferative programs (e.g. DDR inhibition, mitotic death, autophagic death, senescence) appear to operate in concert as a multi-tiered cell-intrinsic safeguard against genome instability. Whether this clear parallel with the role of STING-driven inflammatory signalling as an immune-dependent genome surveillance mechanism [8, 9, 12] alludes to a primordial function for non-immune cell-intrinsic effects in genome surveillance remains unclear [61]. Whether and how genome surveillance mechanisms, which are primarily described to restrict the propagation of neoplastic cells, may benefit chromosomally unstable cancers also remain outstanding questions.

Cancer cells are proposed to exist within a narrow optimal range of chromosome mis-segregation rates, above which CIN becomes tumour suppressive [175–181]: a notion that is supported by observations that CIN and aneuploidy render tumour cells more vulnerable to treatments that elevate chromosome mis-segregation rates [182–185]. Given the emerging links between cGAS–STING and CIN in tumours [21, 114], it is tempting to speculate that by emphasising the CIN- and CIN-associated stress-limiting properties of cGAS–STING activation, chromosomally unstable cancers help maintain a window of CIN that is optimal to their survival. However, ascertaining to what extent this notion holds true or not will require further investigation.

## Perspectives

- Chromosomal instability is a key mechanism by which cancers gain resistance to therapy and promote metastasis. A paradox is identified whereby chromosomal instability constitutively activates cGAS–STING signalling — an innate immune pathway critical to immune surveillance.
- Emerging evidence supports context-dependent pro-tumorigenic effects of cGAS–STING signalling, both on a cell-intrinsic and tumour microenvironment level.
- Leveraging our understanding of the mechanisms of cancer-mediated cGAS–STING pathway control and how cGAS–STING signalling can be beneficial to the tumour could identify novel therapeutic targets in chromosomally unstable cancer.

## Open Access

Open access for this article was enabled by the participation of University of Oxford in an all-inclusive *Read & Publish* agreement with Portland Press and the Biochemical Society under a transformative agreement with JISC.

## Abbreviations

CIN: chromosomal instability
DDR: DNA damage response
DSB: double-strand break
EMT: epithelial–mesenchymal transition
HR: homologous recombination
IRDS: IFN-related DNA damage resistance signature
IRF3: interferon regulatory factor 3
ISGs: interferon-stimulated genes
MDSC: myeloid-derived suppressor cell
MN: micronuclei
SASP: senescence-associated secretory phenotype
STING: stimulator of interferon genes
TBK1: TANK-binding kinase 1
TCGA: The Cancer Genome Atlas
TME: tumour microenvironment
TNBC: triple-negative breast cancer
